# DRUG-RELATED PROBLEMS IN CARDIAC NEONATES UNDER INTENSIVE CARE

**DOI:** 10.1590/1984-0462/2020/38/2018134

**Published:** 2020-01-13

**Authors:** Amanda Roseane Farias do Nascimento, Ramon Weyler Duarte Leopoldino, Marco Edoardo Tavares dos Santos, Tatiana Xavier da Costa, Rand Randall Martins

**Affiliations:** a Universidade Federal do Rio Grande do Norte, Natal, RN, Brazil.

**Keywords:** Drug-related side effects and adverse reactions, Medication errors, Infant, newborn, Heart diseases, Efeitos colaterais e reações adversas relacionados a medicamentos, Erros de medicação, Recém-nascido, Cardiopatias

## Abstract

**Objective:**

To determine the frequency and nature of the Drug Related Problems (DRP) in neonates with cardiac diseases admitted to an Intensive Care Unit.

**Methods:**

This prospective cross-sectional study was developed at the Neonatal Intensive Care Unit (NICU) of a teaching maternity hospital in Brazil from January 2014 to December 2016. All neonates diagnosed with any heart disease (congenital heart disease, cardiomyopathy, arrhythmias, etc.) and who were admitted to the NICU for more than 24 hours with at least one prescribed drug were included in the study. Demographic and clinical data were collected from the records of the institution’s clinical pharmacy service. DRP and their respective interventions were independently reviewed and classified by two pharmacists. DRP classification was performed through the Pharmaceutical Care Network Europe v6.2 system.

**Results:**

122 neonates were included in the study. The frequency of neonates exposed to DRP was 76.4% (confidence interval of 95% [95%CI] 65.9–82.0), with a mean of 3.2±3.8 cases/patient. In total, 390 DRP were identified, of which 49.0% were related to “treatment effectiveness”, 46.7% to “adverse reactions” and 1.0% to “treatment costs”. The medicines most involved in DRP were Vancomycin (10.2%; n=46), Meropenem (8.0%; n=36) and Furosemide (7.1%; n=32). Pharmacists performed 331 interventions, of which 92.1% were accepted by physicians and nurses.

**Conclusions:**

The study showed that DRP are very frequent in patients with cardiac diseases hospitalized in the NICU, predominating problems related to the effectiveness and safety of the drug treatment.

## RESUMO

**Objetivo:**

Determinar a frequência e a natureza dos problemas relacionados a medicamentos (PRMs) em neonatos cardiopatas internados em uma unidade de terapia intensiva.

**Métodos:**

Trata-se de um estudo transversal prospectivo desenvolvido na Unidade de Terapia Intensiva Neonatal (UTIN) de uma maternidade de ensino do Brasil, de janeiro de 2014 a dezembro de 2016. Todos os neonatos diagnosticados com alguma doença cardíaca (cardiopatias congênitas, cardiomiopatias, arritmias etc.) e internados na UTIN por período superior a 24 horas, com pelo menos um medicamento prescrito, foram incluídos no estudo. Dados demográficos e clínicos foram coletados a partir dos registros do serviço de farmácia clínica da instituição. Os PRMs e suas respectivas intervenções foram revisadas e classificadas independentemente por dois farmacêuticos. A classificação dos PRMs foi realizada por meio do sistema Pharmaceutical Care Network Europe versão 6.2.

**Resultados:**

Cento e vinte e dois neonatos foram incluídos no estudo. A frequência de neonatos expostos a PRM foi de 76,4% (intervalo de confiança de 95% [IC95%] 65,9–82,0), com média de 3,2±3,8 casos por paciente. Ao todo, 390 PRM foram identificados, sendo que 49,0% estiveram relacionados à “efetividade do tratamento”, 46,7% a “reações adversas” e 1,0% a “custos do tratamento”. Os medicamentos mais envolvidos em PRM foram: vancomicina (10,2%; n=46), meropenem (8,0%; n=36) e furosemida (7,1%; n=32). Os farmacêuticos realizaram 331 intervenções, sendo 92,1% aceitas por médicos e enfermeiros.

**Conclusões:**

O estudo mostrou que PRMs são muito frequentes em pacientes cardiopatas internados em UTIN, predominando problemas relacionados à efetividade e segurança do tratamento medicamentoso.

## INTRODUCTION

Due to the complexity of drug treatments, critically ill patients are prone to drug-related problems (DRPs).^1^ According to the Pharmaceutical Care Network Europe (PCNE), DRP is any event that interferes with a patient’s pharmacotherapy and, consequently, leads to or possible leads to undesirable clinical outcomes.^2^ This definition involves both problems arising from errors in the therapeutic process (prescription, dispensing, administration) as well as the harmful and unexpected effects of the medicine.^3^

In neonates, physiological immaturity, weight changes and off-label drug use contribute to the occurrence of DRPs.^4,5^ The pharmacokinetic and pharmacodynamic peculiarities of neonates are associated with the rapid maturation of organs and systems over time, and the varying degrees of interindividual maturation. Such characteristics may lead to the sudden reach of sub or supra-therapeutic doses and make medication monitoring difficult.^5,6^ Due to the increased need for dose calculations, weight changes in pediatric patients may also impact drug safety.^7^ Another important factor is the widespread use of off-label medicines. Because people do not know how to use them correctly or know about their harmful effects, these drugs are very likely to cause DRPs.^4^

DRPs, when unresolved, can aggravate patients’ clinical conditions, prolonging hospitalization and even leading to death.^8,9^ In the Neonatal Intensive Care Unit (NICU), there are diseases such as infections, heart disease and respiratory problems, which require complex medical interventions,^10^ highlighting the lack of studies on DRP in this population, especially in patients with heart diseases. A study conducted in an Egyptian hospital of children with heart disease, estimated that the prevalence of DRP in this group was 100%.^11^ Heart disease stands out among the most lethal diseases in the NICU. In Brazil, neonatal mortality related to heart disease was 336 deaths per 1,000 inpatients in 2016, a value six times higher than the overall proportion of deaths in hospitalized neonates.^12^

Given this, DRPs are believed to be quite frequent in neonates with heart disease. Thus, the aim of this study was to determine the frequency and nature of DRPs in neonates with heart diseases admitted to a NICU.

## METHOD

This was a cross-sectional study, with prospective data collection, developed at the NICU of a high-risk pregnancy reference maternity hospital in the state of Rio Grande do Norte, Brazil. All neonates diagnosed with heart disease (congenital heart disease, cardiomyopathy, arrhythmias, etc.) admitted to the NICU between January 2014 and December 2016 for more than 24 hours and on at least one prescribed drug were included in the study. The study excluded neonates who had only been prescribed electrolyte solutions, parenteral nutrition, blood and blood products, oxygen therapy, diagnostic agents and vitamin and mineral supplements, which were not considered medicines.

Data collection was performed from the records of the institution’s clinical pharmacy service, in which the following variables were observed: gender, gestational age, birth weight, length of stay in the NICU, and type of heart disease. During their stay in the NICU, the newborns were evaluated regarding the number of drugs prescribed, the occurrence of DRP and their interventions.

The identification of DRPs was performed by the NICU clinical pharmacy team, consisting of a chief pharmacist and four resident pharmacists. They reviewed nursing records, prescriptions and reports. Two pharmacists (RWDL and METS), had supporting materials such as the Neofax^®^ 2011 book^13^ and the Micromedex^14^ and Uptodate^®^,^15^ databases. They independently reviewed and classified the DRPs. In the case of a disagreement between the evaluators, a third pharmacist (TXC) was consulted. Clinical pharmacists performed the intervention on DRPs, and these interventions were consensually reviewed by the evaluators while other health professionals rated them for acceptance.

The DRPs were classified according to the PCNE system version 6.2, ^2^ by problems and causes. The problems were grouped into: treatment effectiveness, when the drug did not or may not have had the expected effect; adverse reaction, when the patient suffered or was at high risk of an adverse drug event; and the cost of treatment, when other cost-effective medications were available for treatment. DRPs that did not fit into any of these three categories were classified as problems that had no defined category. The categories of causes were: drug selection, when the cause of DRP was related to improper drug selection; dose selection when the cause of DRP was related to the inadequate choice of dose or regimen of the drug; medication use process, when the cause of DRP was related to the wrong administration of the medication; and logistical errors, when the cause of DRP was related to prescription errors, a lack of necessary information, and medication dispensing.

Data were descriptively analyzed by mean and standard deviation for continuous and discrete variables; analyzed by absolute and relative frequency for binary variables; and analyzed by median and interquartile range for the length of hospitalization variable. DRP exposure data were presented in percentage estimates and 95% confidence intervals (95%CI). For each drug, the risk associated with DRP and its 95%CI was calculated, and was defined as the number of times the drug was involved in a DRP, divided by the number of times it was prescribed. Pearson’s chi-square test was also applied to evaluate the association between DRP and mortality. Statistical analysis was performed with Stata^®^ 11 (Stata Corporation, College Station, TX, USA).

This study was approved by the Research Ethics Committee of the Onofre Lopes University Hospital, under protocol No. 580.201/2014.

## RESULTS

The study included 122 neonates with heart disease whose main demographic characteristics were (Table 1): 54.6% males, mean gestational age of 33.1 ± 5.1 weeks and mean birth weight of 2,084 ± 1,019 g. The median length of hospitalization was 30 days (range: 1–278 days) and each patient received an average of 12.4 ± 8.0 medications. During the study, 18.8% of the neonates died, but there was no statistically significant difference in the deaths between the group with and without DRPs (17.6 versus 22.6%; p = 0.539).

**Table 1:** Demographic and clinical characteristics of the study population (n=122).

Characteristics	Value	
Gestational age in weeks (mean, SD)	33.1	5.1
Male, n (%)	65	54.6
Birth weight in grams (mean, SD)	2,084	1,019
Length of hospitalization in days (median, IQR)	30	1–278
Type of heart disease (n,%)		
Persistence of ductus arteriosus	37	35.6
Communication between heart chambers	23	22.1
Pulmonary artery atresia/stenosis	11	10.6
Heart valve defect	10	9.6
Aortic coarctation	9	8.7
Transposition of the great vessels	7	6.7
Cardiomyopathies	4	3.9
Arrhythmias	3	2.8
Drugs used (average, SD)	12.4	8.0
Deaths (n,%)	23	18.8

SD: standard deviation; IQR: Interquartile range.

Overall, 390 DRPs were identified. These problems affected 74.6% (95%CI 65.9–82.0) of the neonates, with an average of 3.2 ± 3.8 cases per patient. The most common problems were “treatment effectiveness” (49.0%) and “adverse reaction” (46.7%), which affected 53.3 and 54.3% of the neonates, respectively. Thirteen DRPs did not fall into any of the PCNE system categories. An example of this were the problems related to errors in meropenem prescriptions due to the lack of information on drug reconstitution and dilution (Table 2).

**Table 2:** Profile of drug related problems identified according to the Pharmaceutical Care Network Europe version 6.2 classification.

Profile (n = 390)	Value	
DRP patients (n, % [95%CI])	91	74.6 (65.9–82.0)
DRP per patient (mean, SD)	3.2	3.8
DRP by type (n [%],% of patients exposed)		
Treatment effectiveness	191 (49.0)	53.3 (65/122)
Adverse effect	182 (46.7)	54.4 (70/122)
Cost of treatment	4 (1.0)	2.4 (3/122)
Uncategorized problem defined	13 (3.3)	9.8 (12/122)

DRP: drug related problems; 95%CI: 95% confidence interval; SD: standard deviation.

The main cause of DRP was “medication use process” (32.6%), with an emphasis on “medication administered incorrectly” (18.2%) and “inappropriate administration time and/ or interval” (7.4%). Another frequent cause was “dose selection” (30.8%), represented mainly by “very low drug dose” (13.1%) and “very high drug dose” (8.2%). Table 3 details the main causes of DRP.

**Table 3:** Profile of drug related problems identified according to the Pharmaceutical Care Network Europe version 6.2 classification.

Causes (n = 390)	n	%
Drug use process		
Mistakenly administered drug	71	18.2
Inappropriate administration time/interval	29	7.4
Too much of the drug was administered	15	3.9
Drug not fully administered	12	3.1
Total	127	32.6
Dose selection		
Very low drug dose	51	13.1
Very high drug dose	32	8.2
Insufficient dosage regimen	19	4.9
Disease evolution requiring dose adjustment	16	4.1
Very frequent dosing regimen	2	0.5
Total	120	30.8
Logistical errors		
Prescription error (lack of required information)	59	15.1
Prescription drug unavailable	18	4.6
Total	77	19.7
Drug selection		
Drug interaction	25	6.4
Inappropriate drug	1	0.3
Drug without indication	1	0.3
Necessary and not prescribed drug	1	0.3
Total	28	7.2

Table 4 shows the medications most involved in DRP, distributed by their general prescription frequencies and risk associated with DRP. The patients received a total of 1,516 medications, with DRPs occurring 449 times. The most commonly prescribed drugs were gentamicin (6.1%), furosemide (5.2%) and dobutamine (4.8%); and those frequently related to DRP were vancomycin (10.2%), meropenem (8.0%) and furosemide (7.1%). It is worth noting that vancomycin was the drug associated with the highest risk for DRP.

**Table 4:** Distribution of top ten drugs involved in drug-related problems by overall prescription frequency and associated risk.

Drugs	Cases		General prescription		Risk (95%CI)
	n	%	n	%	
Vancomycin	46	10.2	42	2.8	1.10 (1.00–1.16)
Meropenem	36	8.0	55	3.6	0.65 (0.60–0.71)
Furosemide	32	7.1	79	5.2	0.41 (0.37–0.45)
Gentamicin	28	6.2	92	6.1	0.30 (0.27–0.34)
Dobutamine	23	5.1	73	4.8	0.32 (0.22–0.45)
Amphotericin	22	4.9	22	1.5	1.00 (0.82–1.20)
Alprostadil	21	4.7	35	2.3	0.65 (0.46–0.77)
Amikacin	21	4.7	65	4.3	0.32 (0.01–0.85)
Aminophylline	17	3.9	46	3.0	0.37 (0.01–0.99)
Captopril	16	3.6	24	1.6	0.67 (0.03–1.39)
Total	449	100.0	1,516	100.0	

95%CI: 95% Confidence Interval.

All in all, pharmacists intervened in 331 (84.9%) DRPs. Of the 331 interventions, 203 were “recommendations for doctors” and 128 were “recommendations for nurses”, of which 92.6% (188/203) and 91.4% (117/128) were accepted, respectively. The overall acceptance ratio was 92.1% (305/331).

## DISCUSSION

The main finding of the study was the high frequency of newborns with heart disease who were exposed to DRP. There was an emphasis on “treatment effectiveness” problems, which were usually caused by the “drug use process”. In addition, the drugs most involved in DRP were vancomycin, meropenem and furosemide, and most pharmaceutical interventions were accepted by the NICU staff. These findings provide enlightening information for health professionals, and contribute to the development of strategies for the prevention and reduction of DRP.

Over three years of observation at the NICU, 75% of infants with heart disease experienced at least one DRP. This result was higher than what has been found in international and Brazilian studies conducted in general pediatrics, in which DRP ranged from 20 to 70%.^16-21^ The result, however, is lower than what was observed in the study by Sabry et al.,^11^ in which all 60 cardiopathic children evaluated were exposed to DRP while in an Egyptian hospital. However, it is worth noting that local specificities, population characteristics, methods of identification and classification of DRPs and the follow-up time of the study make these comparisons difficult.

Problems related to the effectiveness and safety of drug treatments are quite common in the NICU. The reasons for the recurrence of such problems are the rapid changes in body weight and body surface and the maturation of neonates’ organs and systems, which leads to the constant need for dose adjustments. Pharmacokinetics change and, consequently, there is a need for greater care in the prescription and administration of medications in this population.^5,6^ In addition, these problems are accentuated by the lack of appropriate medications for the pediatric population.^22,23^

Similar to pediatric studies, antimicrobials were the group of drugs most involved in GRP.^16-20,24,25^ Some authors state that the risk of adverse events related to each drug is directly associated with the frequency of prescription.^24,25^ However, similar to the findings of Blix et al.,^26^ in our study the most involved medications were not necessarily the most prescribed, showing that the risk of damage is related to the chemical and pharmacological characteristics of the medications, as well as patient characteristics. It is worth noting that vancomycin was the drug associated with the highest risk of DRP due to the frequent need for dose adjustment according to age and renal function. Furthermore, there are minimal infusion requirements due to severe infusion-associated adverse reactions due to rapid infusion (for example, red man syndrome).^27,28^

Pharmacists intervened in 17 out of every 20 DRPs identified, and virtually all of the proposed interventions were accepted by doctors and nurses, a finding also seen in other studies.^18,19,21^ Thus, the broad support provided by the NICU professional team demonstrates the need to strengthen clinical pharmacy services as one of the main strategies for prevention and reduction of DRP.

The limitations of the study include the fact that it deals with secondary data collected from a non-probabilistic sample of a single institution and the indirect observation of administration errors, which may have led to the underestimation of these problems. However, the findings of the study are strengthened by its prospective nature and the fact that it adopted an internationally recognized DRP classification system.

Thus, this study showed that DRPs are very common in patients with cardiac diseases hospitalized in NICUs. The main problems were related to treatment effectiveness, principally due to the inappropriate process of drug use. The drugs most involved in DRP were vancomycin, meropenem and furosemide. In general, most pharmaceutical interventions were performed with the primary collaboration of physicians and had an overall acceptance rate exceeding 90%. Finally, further research on the subject is needed, involving clinical outcomes associated with DRP, as well as studies that help improve drug treatment, like instruments that facilitate the prescription, dispensation and/or administration of medications.

## References

[1] Raju TN, Suresh G, Higgins RD (2011). Patient Safety in the context of Neonatal Intensive Care: research and educational opportunities. Pediatr Res.

[2] Pharmaceutical Care Network Europe [homepage on the Internet] (2010). Classification of drug related problems (revised 14-01-2010vm).

[3] van den Bemt PM, Egberts TC, de Jong-van den Berg LT, Brouwers JR (2000). Drug-related problems in hospitalised patients. Drug Saf.

[4] Sorrentino E, Alegiani C (2012). Medication errors in the neonate. J Matern Fetal Neonatal Med.

[5] Rodieux F, Wilbaux M, van den Anker JN, Pfister M (2015). Effect of kidney function on drug kinetics and dosing in neonates, infants, and children. Clin Pharmacokinet.

[6] Wilbaux M, Fuchs A, Samardzic J, Rodieux F, Csajka C, Allegaert K (2016). Pharmacometric approaches to personalize use of primarily renally eliminated antibiotics in preterm and term neonates. J Clin Pharmacol.

[7] Chua SS, Chua HM, Omar A (2010). Drug administration errors in paediatric wards: a direct observation approach. Eur J Pediatr.

[8] Kunac DI, Kennedy J, Austin N, Reith D (2009). Incidence, preventability, and impact of Adverse Drug Events (ADEs) and potential ADEs in hospitalized children in New Zealand: a prospective observational cohort study. Paediatr Drugs.

[9] Ohta Y, Sakuma M, Koike K, Bates DW, Morimoto T (2014). Influence of adverse drug events on morbidity and mortality in intensive care units: the JADE study. Int J Qual Health Care.

[10] Hedstrom A, Ryman T, Otai C, Nyonyintono J, McAdams RM, Lester D (2014). Demographics, clinical characteristics and neonatal outcomes in a rural Ugandan NICU. BMC Pregnancy Childbirth.

[11] Sabry NA, Farid SF, Dawoud D (2016). Drug related problems in cardiac children. Minerva Pediatr.

[12] Brazil – Ministério da Saúde (2019). Uma análise da situação de saúde e das doenças e agravos crônicos: desafios e perspectivas. Brasília (DF): Ministério da Saúde.

[13] (2010). Neofax^®^2010 – Manual of Drugs Used in Neonatal Care.

[14] (2017). Uptodate^®^ [homepage on the Internet].

[15] (2017). Micromedex Solutions® Healthcare Series of Database Versão 2.0 [homepage on the Internet].

[16] Rashed AN, Neubert A, Tomlin S, Jackman J, Alhamdan H, AlShaikh A (2012). Epidemiology and potential associated risk factors of drug-related problems in hospitalised children in the United Kingdom and Saudi Arabia. Eur J Clin Pharmacol.

[17] Rashed AN, Wilton L, Lo CC, Kwong BY, Leung S, Wong IC (2014). Epidemiology and potential risk factors of drug-related problems in Hong Kong paediatric wards. Br J Clin Pharmacol.

[18] Ibrahim N, Wong IC, Tomlin S, Sinha MD, Rees L, Jani Y (2015). Epidemiology of medication-related problems in children with kidney disease. Pediatr Nephr.

[19] Okumura LM, Silva DM, Comarella L (2016). Relation between safe use of medicines and Clinical Pharmacy Services at Pediatric Intensive Care Units. Rev Paul Pediatr.

[20] Birrara MK, Heye TB, Shibeshi W (2017). Assessment of drug-related problems in pediatric ward of Zewditu Memorial Referral Hospital, Addis Ababa, Ethiopia. Int J Clin Pharm.

[21] Malfará M, Pernassi M, Aragon D, Carlotti A (2018). Impact of the clinical pharmacist interventions on prevention of pharmacotherapy related problems in the paediatric intensive care unit. Int J Clin Pharm.

[22] Chappell K, Newman C (2004). Potential tenfold drug overdoses on a neonatal unit. Arch Dis Child Fetal Neonatal.

[23] Schweigertova J, Durisova A, Dolnikova D, Ondriasova E, Balazova M, Slezakova V (2016). Off-label and unlicensed use of medicinal products in the neonatal setting in the Slovak Republic. Pediatr Int.

[24] Stavroudis TA, Shore AD, Morlock L, Hicks RW, Bundy D, Miller MR (2010). NICU medication errors: identifying a risk profile for medication errors in the neonatal intensive care unit. J Perinatol.

[25] Pawluk S, Jaam M, Hazi F, Al Hail MS, El Kassem W, Khalifa H (2017). A description of medication errors reported by pharmacists in a neonatal intensive care unit. Int J Clin Pharm.

[26] Blix HS, Viktill KK, Reikvam A, Moger TA, Hjemaas BJ, Pretsch P (2004). The majority of hospitalised patients have drug-related problems: results from a prospective study in general hospitals. Eur J Clin Pharmacol.

[27] Benner KW, Worthington MA, Kimberlin DW, Hill K, Buckley K, Tofil NM (2009). Correlation of vancomycin dosing to serum concentrations in pediatric patients: a retrospective database review. J Pediatr Pharmacol Ther.

[28] Bauters T, Claus B, Schelstraete P, Robays H, Benoit Y, Dhooge C (2012). Vancomycin-induced red man syndrome in pediatric oncology: still an issue?. Int J Clin Pharm.

